# Deep Learning-Based CT Imaging for the Diagnosis of Liver Tumor

**DOI:** 10.1155/2022/3045370

**Published:** 2022-06-16

**Authors:** Heng Zhang, Kaiwen Luo, Ren Deng, Shenglin Li, Shukai Duan

**Affiliations:** ^1^College of Mathematics and Statistics, Southwest University, Chongqing 400715, China; ^2^Department of Military Logistics, Army Logistic University of PLA, Chongqing 401331, China; ^3^College of Artificial Intelligence, Southwest University, Chongqing 400715, China

## Abstract

The objective of this research was to investigate the application value of deep learning-based computed tomography (CT) images in the diagnosis of liver tumors. Fifty-eight patients with liver tumors were selected, and their CT images were segmented using a convolutional neural network (CNN) algorithm. The segmentation results were quantitatively evaluated using the Dice similarity coefficient (DSC), precision, and recall. All the patients were examined and diagnosed by CT enhanced delayed scan technique, and the CT scan results were compared with the pathological findings. The results showed that the DSC, precision, and recall of the CNN algorithm reached 0.987, 0.967, and 0.954, respectively. The images segmented by the CNN were clearer. The diagnostic result of the examination on 56 cases by CT enhanced delay scanning was consistent with that of pathological diagnosis. According to the result of pathological diagnosis, there were 6 cases with hepatic cyst, 9 with hepatic hemangioma, 12 cases with liver metastasis, 10 cases with hepatoblastoma, 3 cases with focal nodular hyperplasia, and 18 cases with primary liver cancer. The result of CT enhanced delay scanning on 58 patients was consistent with that of pathological diagnosis, and the total diagnostic coincidence rate reached 96.55%. In conclusion, the CNN algorithm can perform accurate and efficient segmentation, with high resolution, providing a more scientific basis for the segmentation of liver tumors in CT images. CT enhanced scanning technology has a good effect on the diagnosis and differentiation of liver tumor patients, with high diagnostic coincidence rate. It has important value for the diagnosis of liver tumor and is worthy of clinical application.

## 1. Introduction

The liver is located in the right upper abdomen of the human body and is the largest parenchymal organ in the abdominal cavity, accounting for about 2% of the body weight [1]. As an important digestive organ of the human body, the liver has complex functions such as deoxidation, storage of liver sugar, immunity, blood coagulation, and bile generation and excretion [2]. The liver also has the functions of detoxification, metabolism, regeneration, and immune defense. It can decompose the protein, fat, carbohydrate, and other nutrients absorbed by the human body and synthesize the important substances needed for human metabolism. The liver has a special blood supply system for hematopoiesis, blood storage, and regulation of circulating blood volume, and the blood circulation system has a complex structure. It is the only organ in the human body with double blood supply systems from hepatic artery and portal vein and the only organ for albumin synthesis [3]. The liver undertakes various important metabolic functions in the human body and is also one of the organs where tumors are easy to grow, among which malignant tumors are more frequent. Liver cancer is a common malignant tumor, and it is the second leading cause of cancer death in China [4]. According to Global Cancer Statistics released by the WHO, about 840,000 people were newly diagnosed with malignant hepatic tumor and 780,000 people died of it in 2018. The malignant hepatic tumor grows rapidly and is undetectable at the initial stage [5]. In addition, blood supply is abundant in the liver, involving many important organs. Primary liver cancer is the common malignant tumor in the human gastrointestinal tract. In recent years, the incidence of domestic primary liver cancer grows year by year, accounting for 13.13% among all cancers. Most of the patients with primary liver cancer are young. According to domestic cancer statistics in 2017, the incidence of primary liver cancer is ranked in the 4^th^ place with the mortality following only lung cancer and gastric cancer. Relevant statistical data demonstrate that the 5-year survival rate of primary liver cancer patients without systematic treatment is lower than 5%. In contrast, that of primary liver cancer patients receiving systematic treatment is increased to 35% [6, 7].

China has a large population base, and the risk factors leading to liver cancer are numerous and complex. The WHO points out that about 30% of cancer diseases can be prevented [8], so it is necessary to check the high-risk population for liver cancer. Early diagnosis and early treatment are important links to save liver cancer patients. Early diagnosis of liver tumors mainly uses imaging diagnosis, molecular marker detection, and protein marker detection [9]. Imaging examination is a convenient and rapid method for large-scale screening. Computed tomography (CT) imaging technique can solve the fuzzy problem leading to poor contrast and unidentifiable structure of X-ray. Its resolution for high-density tissue is clear and can well display the blood vessels and vascular lesions. It can also realize the three-dimensional imaging to show tissue and organs and highlight the diseased organs. Therefore, CT imaging has the characteristics of high spatial resolution, fast imaging speed, and moderate price, making it one of the commonly used examination methods for liver disease and widely used in the diagnosis of liver disease [10]. At present, liver tumor resection is still the most effective means to completely cure liver cancer [11]. Accurate and reliable segmentation of the liver from abdominal CT images is a very important step in the early diagnosis of liver diseases. Then, from the liver organ, canceration area is accurately segmented. In clinical applications, the segmentation of liver and tumor tissue mainly relies on rich practical experience of doctors from manual segmentation. However, the segmentation process is not only time- and energy-consuming, it is largely affected by subjective factors such as doctors' past experience, so different doctors often get different results. Due to the heterogeneity and diffusivity of tumor morphology, automatic segmentation of tumor lesions is very challenging.

Computer technology is constantly developed and widely applied in medical imaging. Deep learning can accomplish the segmentation of lesions only by inputting original data to avoid the limitation and low efficiency of image information acquisition caused by artificial feature segmentation [12]. As a deep learning module, convolutional neural network (CNN) algorithm possesses the characteristics of the automatic segmentation algorithm. Deep learning is used to segment 51 CT images of liver tumors, including 30 scans for training and 21 for control. Dice similarity coefficient (DSC) and relative volume difference (RVD) are adopted to evaluate the segmentation quality of the images processed by deep learning. The indexes are 83.2 ± 7.8% and 18.6 ± 17.4%. Compared with the use of U-Net or LOGISMOS alone, the two indexes are both remarkably improved (*p* *<* 0.05) [13]. The images processed by deep learning can enhance the diagnostic accuracy and efficiency of liver tumors, showing the positive application values. The research purpose is to investigate the application of CT imaging in diagnosing liver tumor by the segmentation of the lesions in liver tumor CT imaging with the CNN algorithm. Furthermore, an effective reference is provided for the efficient application CT imaging in the clinical diagnosis of liver tumor.

## 2. Materials and Methods

### 2.1. Research Subjects

In this study, a total of 58 patients admitted to hospital were selected as the research subjects, including 30 male patients and 28 female patients, aged from 39 to 76, with a mean of 57.5 ± 2.7 years. There was no statistically significant difference (*p* *>* 0.05) in general basic information such as age, gender, body mass index, and condition for all patients in this study. All patients and their families had signed informed consent, and this study had been approved by the ethics committee of the hospital.

Inclusion criteria are as follows: (1) Aged between 39 and 68 years; (2) Patients diagnosed as liver tumor according to pathological diagnosis; and (3) All clinical information of the patient was complete.

Exclusion criteria are as follows: (1) Aged beyond 39∼68; (2) There are other serious complications; (3) Allergic to contrast media and sterilized alcohol; and (4) With mental problems or consciousness disorders, poor compliance.

### 2.2. CT Scanning Methods

The scanning equipment is a 64-row CT scanner. During CT scanning, patients were told to fast within 12 hours in advance, and 500 mL of 1%∼2% meglumine diatrizoate solution was given before clinical examination. The scanning range was from the patient's diaphragm to L3, and the scanning time was 12∼23 s. After scanning, continuous cross-sectional reconstruction was performed with a slice thickness of 5∼10 mm. A CT plain scan was given first, and then the CT enhanced delayed scan was performed. Then, 80 mL of contrast agent was injected intravenously for delayed scanning at 2∼3 mL/min. The scanning process was from the arterial phase to venous phase, and finally to the delayed phase. Images of the lesion were formed through a thin layer of 5∼10 mm, and then maximum density projection and multiplanar reconstruction were performed.

### 2.3. Automatic Image Segmentation Algorithm Based on CNN

**Figure 1:**
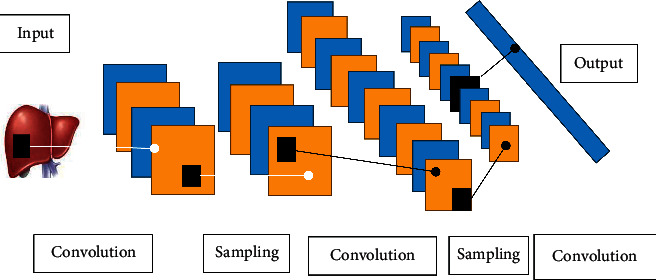
The basic structure of CNN.

The CNN automatic image segmentation algorithm has a multilayer network structure which is similar to the traditional neural network algorithm model and can be reversed. It mainly includes the input layer, convolution layer, pooling layer, connection layer, and output layer [14]. This algorithm is a supervised deep learning method and has achieved great success in image recognition, speech recognition, and natural language processing. CNN has been widely used in the field of medical image processing, including detection of breast cancer and cell cancer and brain lesions, knee cartilage segmentation, brain tumor segmentation, and liver tumor segmentation [15, 16]. Compared with the traditional neural network structure, the input of CNN is usually the images, and the network structure can be modified according to the input image data with certain spatial characteristics, so as to maximize the utilization of the input image data, reduce the setting of other auxiliary parameters, and speed up the operation of the neural network.  Figure 1 shows the flowchart of the CNN image segmentation algorithm.

As a network structure algorithm for automatic image segmentation and recognition, CNN can automatically divide corresponding feature areas without extracting target features in advance. The input layer is usually the image mode, and the function of convolution layer is to extract image features. Its mathematical calculation equation is shown as below:

(1) $$\begin{array}{c} n_{j}^{X}=f\left(\underset{i\in M}{\sum }n_{j}^{X-1}*a_{ij}^{X}+m_{j}^{X}\right), \end{array}$$

where *n*_*j*_^*X*^ indicates the output result; *f* stands for activation function; *n*_*j*_^*X*^ represents the output result of *X* − 1 layer (the previous layer); *∗* stands for the convolution operation; *a*_*ij*_^*X*^ represents the weight of each convolution kernel; and *m*_*j*_^*X*^ represents the offset. The activation functions are used to map the extracted features to a specific space by a nonlinear function, namely, it is to improve the nonlinear action. Activation functions generally include Sigmoid function and Relu function. The Sigmoid function is expressed as equation (2); and the Relu activation function is expressed as (3):

(2) $$\begin{array}{c} \sigma \left(x\right)=\frac{1}{1+e^{-x}}, \end{array}$$

(3) $$\begin{array}{c} R\left(x\right)=\max\left(o,x\right)=\left\{\begin{array}{cc} x, & x\geq 0,\\ 0, & x<0. \end{array}\right) \end{array}$$

### 2.4. Evaluation Indicator of CNN Automatic Segmentation Algorithm

To evaluate whether the CNN automatic segmentation algorithm can accurately segment CT images of liver tumors, DSC, precision, and recall are used for quantitative evaluation. The accuracy rate represents the ratio of the number of positive samples correctly segmented to the number of all positive samples segmented, and a value closer to 1 indicates more accurate segmentation results. The recall rate represents the ratio of the number of positive samples correctly segmented to the number of all positive samples, and a closer value to 1 indicates better segmentation results. A larger Dice similarity coefficient indicates a higher coincidence degree between the segmentation results and the gold standard. The calculation methods of the three indicators are as follows:

(4) $$\begin{array}{c} \text{DSC}=\frac{2\left|A\cap B\right|}{\left|A\right|+\left|B\right|},\\ \text{precision}=\frac{\text{TP}}{\text{TP}+\text{FP}},\\ \text{recall}=\frac{\text{TP}}{\text{TP}+\text{FN}}. \end{array}$$

TP indicates the true positive (segmented true lesion); FP indicates the false positive (false lesion segmented incorrectly); FN indicates the false negative (true lesion that has not been segmented); *A* represents the segmentation results by imaging doctors; and *B* represents the segmentation results of the automatic algorithm.

### 2.5. Observation Indicators

By comparison between CT enhanced delayed scanning results and pathological examination results, the diagnostic accuracy of CT enhanced delayed scanning was analyzed.

### 2.6. Statistical Analysis

In this study, SPSS 20.0 statistical software was used to analyze the data, and the measurement data conforming to normal distribution was analyzed by the one-way variance method, and the count data were tested by *X*^2^ and expressed by percentage. *p* *<* 0.05 indicates that the difference was significant.

## 3. Results

### 3.1. Image Segmentation Result

**Figure 2:**
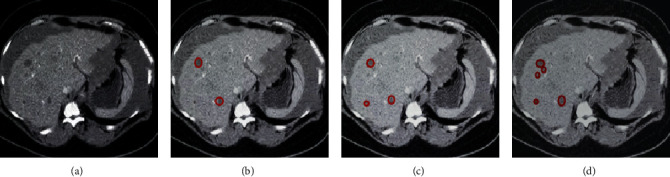
The results of liver tumor image segmentation by different algorithms. (a) The original CT image of the liver tumor. (b) The segmentation results using the HED algorithm. (c) The segmentation results using the U-Net algorithm. (d) The segmentation results using the CNN algorithm.

The CNN automatic segmentation algorithm was used to segment the CT enhanced delayed images of liver tumors, as shown in Figure 2. After that, the segmentation results were compared with the HED [17] algorithm and the U-Net algorithm.  Figure 2(a) shows the original CT image of a patient with liver tumor.  Figure 2(b) shows the segmentation results of the HED algorithm, and Figure 2(c) shows the segmentation results of the U-Net algorithm.  Figure 2(d) shows the lesion segmentation results using the CNN algorithm. It was worth noting that the lesion segmentation accuracy of Figure 2(d) is higher than that of Figures 2(c) and 2(b).

### 3.2. Evaluation Metrics for Segmentation Results

**Figure 3:**
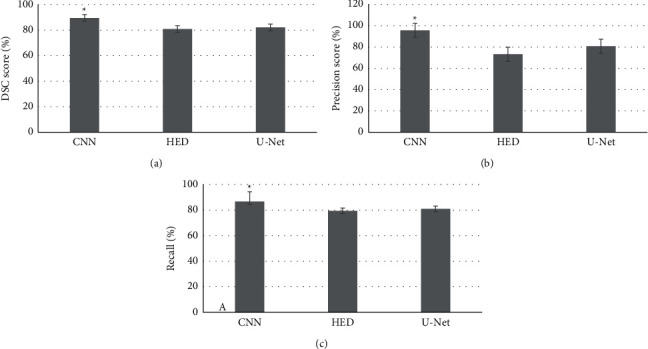
Evaluation indicators of different lesion segmentation algorithms. (a) DSC comparison results of HED algorithm, CNN algorithm, and *U*-Net algorithm. (b) Precision comparison of HED algorithm, CNN algorithm, and *U*-Net algorithm. (c) Recall comparison of HED algorithm, CNN algorithm, and *U*-Net algorithm. ^*∗*^Compared with CNN algorithm and U-Net algorithm, *p* < 0.05.

To quantitatively evaluate the segmentation results, DSC, precision, and recall were used to evaluate the segmentation results of the three algorithms. The algorithm of this work was compared with the *U*-Net algorithm and the HED algorithm. The results showed that the DSC, precision, and recall rates of the algorithm in this work were 89.43%, 95.74%, and 86.68%, respectively; those of the HED algorithm were 80.82%, 73.31%, and 79.46%, respectively; and those of the *U*-Net algorithm were 82.13%, 80.78%, and 80.92%, respectively. Compared with the HED and *U*-Net algorithms, the three indicators of the CNN automatic segmentation algorithm were improved. This showed that the algorithm used in this work had a good segmentation effect on CT images of liver tumors, as shown in Figure 3.

### 3.3. Comparison between CT Scan and Pathological Examination

**Figure 4:**
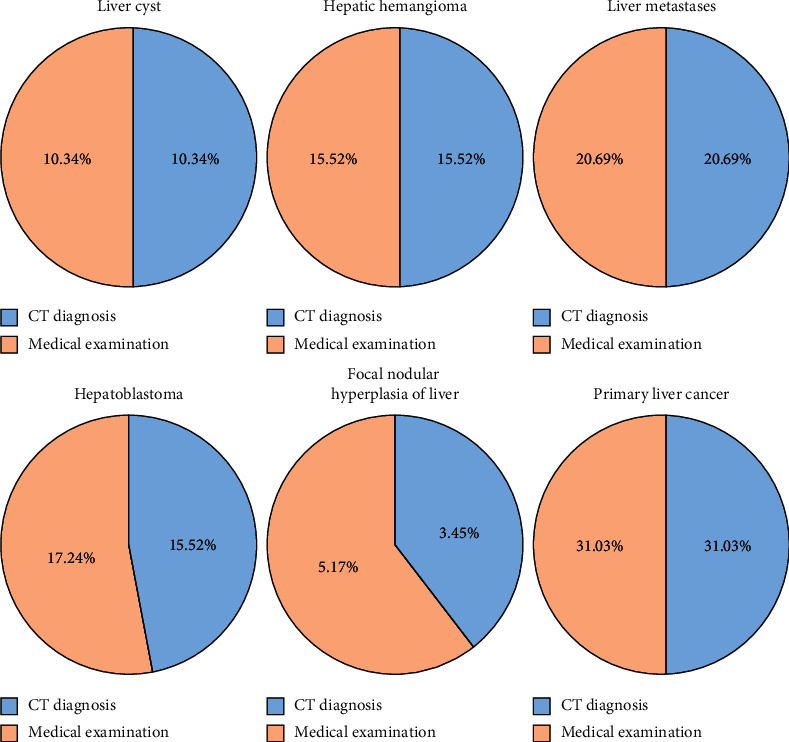
Diagnostic coincidence rate between CT scan and pathological results.

Pathological diagnosis results showed hepatic cysts in 6 cases, hepatic hemangiomas in 9 cases, liver metastases in 12 cases, hepatoblastoma in 10 cases, focal nodular hyperplasia in 3 cases, and primary liver cancer in 18 cases. The CT enhanced delayed scanning results of 58 patients were in accordance with pathological diagnosis results, and the total diagnostic coincidence rate was 96.55%. Two patients were misdiagnosed as hepatoblastoma and focal nodular hyperplasia of liver. Detailed results are shown in Table 1 and Figure 4.

### 3.4. CT Enhanced Scanning Delay Results

After 58 patients received CT enhanced delayed scanning, there were 6 hepatic cysts, accounting for 10.34%, and their CT images showed no enhancement in the arterial phase; there were 9 cases of hepatic hemangioma, accounting for 15.52%. Of the 9 cases, 4 cases were typical, and the edge of CT arterial phase showed nodular or cloud-like enhancement, spreading to the center, and the other 2 cases were atypical; there were 12 cases of liver metastases, accounting for 20.69%, and their CT images showed continuous circular enhancement at the edge of portal vein; there were 10 cases of hepatoblastoma, accounting for 17.24%, and the delayed CT imaging showed a low-density state; there were 3 cases of focal nodular hyperplasia of liver, accounting for 5.17%, and CT imaging showed typical enhancement; and there were 18 cases of primary liver cancer, of whom 15 cases were typical enhancement, and their CT images showed transient nodular enhancement in hepatic artery phase, low-density shadow in the delayed phase. There was no obvious enhancement in the other 3 cases.

## 4. Discussion

The liver is an important organ of the human body and responsible for the detoxification, metabolism, and synthesis processing. At the same time, the liver is also the heaviest organ in the human abdomen, accounting for about 1.5% to 2.5% of the total weight of the human body. The size and shape of the liver vary from person to person. Affected by the environment and poor eating habits, more and more people are suffering from liver diseases such as cirrhosis, liver ascites, and liver cancer. The liver is a common site for the development of primary (originated from cancerous liver cells) or secondary (e.g., spreading from rectal cancer to liver) tumors. Liver cancer is a common malignant tumor, and it is one of the main causes of cancer death in the world. According to the global cancer statistics report released by the WHO [18], about 840,000 people were newly diagnosed with liver malignant tumors in 2018, and 780,000 people died of liver malignant tumors. Malignant liver tumors grow rapidly and are not easily detected at the initial stage. Moreover, the blood supply in liver organs is very rich, involving many important blood vessels. Primary liver cancer is the most common malignant tumor in human digestive tract. In recent years, the incidence of primary liver cancer in China has increased year by year, accounting for 13.13% of all cancers, and the patients tend to be younger. According to the statistics of cancer in China in 2017, the incidence of primary liver cancer is the fourth, and its mortality rate is only third to lung cancer and gastric cancer. Statistics show that the five-year survival rate of patients with primary liver cancer without systematic treatment is less than 5%, while the survival rate of patients with primary liver cancer after systematic treatment can be increased to 35% [19]. At present, the etiology and pathogenesis of primary liver cancer are not clear, and it is generally accepted by the medical community that it may be caused by virus infection, which makes early detection and prevention difficult [20]. If primary liver cancer can be diagnosed as early as possible, it is helpful to carry out systematic treatment, which can improve the quality of life and survival rate of patients.

In terms of the clinical diagnosis, biopsy is the most effective method for the diagnosis of liver space-occupying lesions. However, as an invasive examination, biopsy will inevitably bring physical and mental harm to patients. At the same time, complications often occur, such as bleeding, infection, and cancer cells spreading to other parts of the liver along the sliced probe [21]. Compared with biopsy, enhanced CT is the most commonly used screening method for liver cancer. With the development of medical technology, medical equipment and technology have been improved and updated. CT examination has been widely used and popularized in clinical disease examination and has gradually been recognized as an auxiliary diagnostic method with a high degree of acceptance by patients [22, 23]. With constant updating of the CT enhanced delayed scanning technology, its examination time is reduced, and image definition is elevated; it can continuously collect and scan volume data, and implement spiral scanning of the whole liver involving hepatic artery, hepatic vein, and portal vein. In addition, CT enhanced delayed scanning technology can collect and scan volume data at one time, reduce the missed diagnosis rate, and clearly show the characteristics and conditions of patients' hepatic vessels and liver tumors [24, 25]. Low-dose CT imaging results in the increase in noise. To distinguish the location of lesions clearly, these original images need to be denoised to make them clearer. In medical diagnosis, the results of manual segmentation are inconsistent and very time-consuming. To overcome these problems, many semiautomatic and automatic liver tumor segmentation methods are put forward successively. Deep learning is widely used in the processing of medical images. CNN is an automatic segmentation algorithm in deep learning and is also used to segment tumor lesions. Li et al. (2021) [26] used deep learning to classify and recognize medical images. The experimental result revealed that the accuracy of deep learning in image classification and recognition was over 80% with stable segmentation performance.

This work explored the effect of using the CNN algorithm to segment CT images of liver tumor patients. The results showed that compared with the images segmented by the HED algorithm and *U*-Net algorithm, the images segmented by the CNN algorithm had higher resolution and precision. In addition, the DSC, precision, and recall rates of the CNN algorithm were 89.43%, 95.74%, and 86.68%, respectively, which were significantly higher than those of the HED algorithm and U-Net algorithm (*p* < 0.05). The result demonstrated that the adopted algorithm showed good segmentation effects on CT images of liver tumor. A total of 58 included patients underwent CT delay scan, and the coincidence rate between scan results and physical examination results was 96.55%, which suggested that the technology could be used to diagnose liver tumor diseases with high diagnostic accuracy. The experimental result was generally consistent with that of the previous studies. In addition, the research result indicated that enhanced CT scan possessed higher sensitivity and could perform the qualitative analysis on lesions.

## 5. Conclusion

In this study, CNN was used for feature segmentation of CT images of liver tumor patients. It proved to be accurate and efficient in image segmentation. Meanwhile, the algorithm had high resolution, providing a scientific basis for the segmentation of liver tumor in CT images. Above, CT enhanced scanning technology had a good effect on the diagnosis and differentiation of liver tumor patients, with a high diagnostic coincidence rate. It has important value for the diagnosis of liver tumor and is worthy of clinical application. However, some limitations in the study should be noted. The sample size is small, which will reduce the power of the study. In the follow-up, an expanded sample size is necessary to strengthen the findings of the study.

## Figures and Tables

**Table 1:** CT scan and pathological diagnostic results.

Tumor type	CT scan results	Medical examination results
Liver cyst	6	6
Hepatic hemangioma	9	9
Liver metastases	12	12
Hepatoblastoma	9	10
Focal nodular hyperplasia of liver	2	3
Primary liver cancer	18	18

## Data Availability

The data used to support the findings of this study are available from the corresponding author upon request.
